# Determination of silver(I)-binding sites in canonical B-DNA by NMR spectroscopy

**DOI:** 10.1007/s00775-025-02115-y

**Published:** 2025-05-02

**Authors:** Tabea Lenz, Uroš Javornik, Marian Hebenbrock, Janez Plavec, Jens Müller

**Affiliations:** 1 Institute of Inorganic and Analytical Chemistry, Universität Münster, Corrensstr. 30, 48149 Münster, Germany; 2 Slovenian NMR Centre, National Institute of Chemistry, SI-1000 Ljubljana, Slovenia; 3 Faculty of Chemistry and Chemical Technology, University of Ljubljana, Ljubljana, Slovenia; 4 EN-FIST Center of Excellence, SI-1000 Ljubljana, Slovenia

**Keywords:** CD spectroscopy, NMR spectroscopy, Silver, B-DNA, Metal-mediated base pairs

## Abstract

The interaction of metal ions with nucleic acids was studied by determining the initial binding sites of Ag^+^ ions at unmodified B-DNA by NMR spectroscopy. In particular, NMR spectra were recorded of the Dickerson-Drew dodecamer sequence in the presence of different ratios of Ag^+^ ions to DNA. The data indicate that the coordination of the first three Ag^+^ ions per duplex preferentially takes place inside the B-DNA helix rather than at other possible binding sites such as the negatively charged phosphate backbone and/or the endocyclic N7 position of purine residues. Larger DNA aggregates are formed in the presence of excess Ag^+^ ions, as indicated by the formation of a precipitate and by significant changes in the circular dichroism spectra. As shown by a titration with chloride ions, the Ag^+^ ions are only loosely bound to the nucleic acids and can be released by precipitation of AgCl.

**Graphical Abstract:**

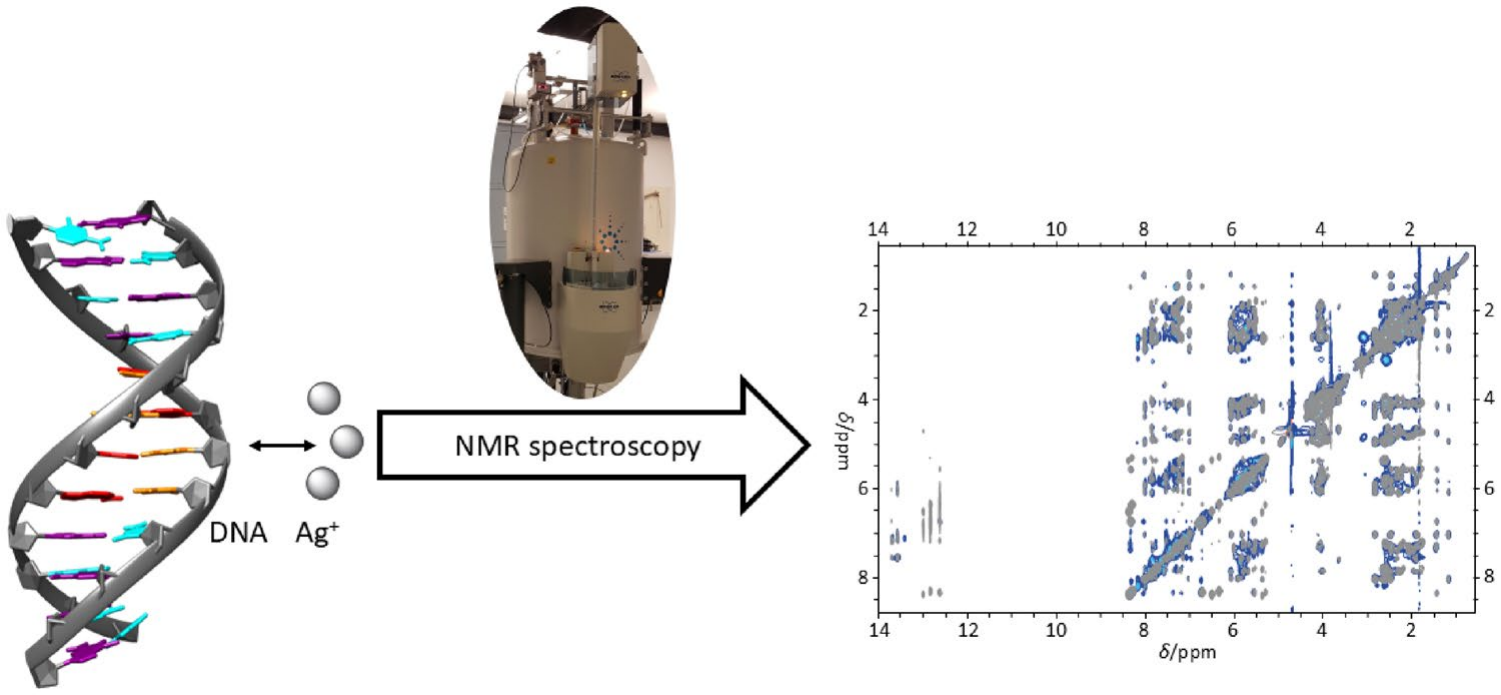

## Introduction

**Fig. 1:**
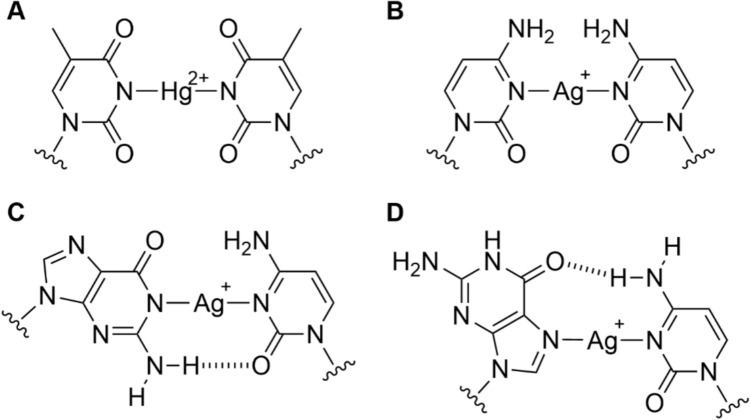
Structural representation of various crystallographically established metal-modified and metal-mediated base pairs: **A** T–Hg^2+^–T [21]; **B** C–Ag^+^–C [17]; **C** G–Ag^+^–C with Ag^+^ binding *via* the Watson-Crick edge [18]; **D** G–Ag^+^–C with Ag^+^ binding *via* the Hoogsteen edge [19]

Nucleic acids provide various positions that can interact with metal ions in different ways: the negatively charged sugar-phosphate backbone is able to coordinate cations *via* electrostatic interactions, making them primary binding sites for hard cations such as alkaline earth ions [1, 2]. The nucleobases in turn contain endocyclic nitrogen atoms, which can coordinate softer cations. The coordination *via* the purine N7 position needs to be emphasized at this point. The purine N7 atom has a high electron density, is – except for Hoogsteen base pairing – not involved in hydrogen bonding, and is easily accessible in the duplex *via* the major groove. These properties make the N7 atom an ideal donor atom for transition metals and hence a likely binding site [3]. Probably the most prominent example of a DNA-binding metal complex is the antitumor drug cisplatin (*cis*-diamminedichloridoplatinum(II)). It binds to the N7 position of purines, preferentially of guanine residues, initiating a variety of cellular responses that eventually lead to apoptotic cell death [4–6]. The coordination of transition metal cations inside the duplex, i.e., along the helical axis, requires the formation of so-called metal-mediated or metal-modified base pairs [7]. In these base pairs, the metal ions are located between two (canonical or artificial) nucleobases of complementary DNA strands [8, 9]. One prominent example involving canonical nucleobases, the Hg^2+^-mediated thymine:thymine pair, was reported already in 1962 (Fig. 1a) [10]. Around the same time, research into the interaction of Ag^+^ ions with nucleic acids was also initiated [11–15]. This work culminated in the discovery of several Ag^+^-mediated base pairs within DNA and RNA duplexes, involving cytosine, guanine, thymine, and for modified DNA also deazapurine residues as ligands (Fig. 1) [16–20].

Interestingly, Ag^+^-mediated base pairs are highly versatile. In the solid state, essentially all canonical nucleobases except for adenine are established as components of Ag^+^-mediated base pairs, namely C–Ag^+^–C, G–Ag^+^–G, T–Ag^+^–T, and G–Ag^+^–C [18, 19]. In solution, A–Ag^+^–C pairs involving adenine were additionally reported [22, 23]. The fact that both homo and hetero base pairs can be mediated by Ag^+^ ions impedes a straightforward design of the oligonucleotide sequence. For example, a sequence designed to contain ten canonical Watson-Crick pairs and two C–Ag^+^–C pairs crystallized as an extended metallo-DNA nanowire of contiguous Ag^+^-mediated base pairs and bulged-out adenine residues [18]. A subsequent study correlated the formation of such duplexes containing contiguous Ag^+^-mediated base pairs with a characteristic pattern in the CD spectrum with entirely negative ellipticity, except for a small positive Cotton effect at ca. 310 nm [19]. Very similar CD spectra were reported for duplexes composed of continuous Ag^+^-mediated homo base pairs of the canonical nucleobases [24–26]. It, therefore, appears obvious that a DNA duplex of any sequence may rearrange into a duplex of contiguous Ag^+^-mediated base pairs, provided that sufficient Ag^+^ ions are available. This poses a challenge for the development of new Ag^+^-mediated base pairs composed of artificial nucleobases with an increased Ag^+^-binding affinity, as these are typically applied as individual pairs embedded into longer stretches of canonical Watson-Crick pairs. In fact, the addition of excess Ag^+^ to duplexes containing such high-affinity binding sites in some cases also leads to the formation of nucleic acids with CD spectra similar to the ones mentioned above [27]. Hence, it is important to investigate the transformation of a regular B-DNA duplex to a duplex with contiguous Ag^+^-mediated base pairs. Towards this end, it is imperative to identify the initial Ag^+^-binding sites on a regular B-DNA duplex. This information will help to understand how B-DNA rearranges into a duplex with contiguous Ag^+^-mediated base pairs.

Early reports from the 1960s on the complexing of DNA by Ag^+^ ions classify three types of binding, based on spectrophotometric and potentiometric experiments: at a low Ag^+^ to nucleobase ratio *r* of < 0.2, a high binding affinity is reported (type I binding). As no protons are released from the DNA at this stage, a binding to the purine N3 or N7 atoms or the formation of a π complex with Ag^+^ being sandwiched between neighbouring nucleobases was suggested [14, 28] and a binding to the phosphate groups was deemed unlikely [11]. With increasing ratio (0.2 < *r* < 0.5), additional binding sites with lower affinity are occupied, accompanied by the release of protons (type II binding). This points towards the formation of metal-modified base pairs. Finally, at ratios >0.5, more complex adducts are formed (type III binding) [11, 14]. Depending on the pH, type I and type II binding may occur consecutively (acidic conditions) or simultaneously (slightly alkaline conditions) [14]. Apparently, DNA rich in G:C pairs is affected to a larger extent by Ag^+^-binding than A:T-rich DNA [15].

The present study investigates for the first time the binding of Ag^+^ ions to a short synthetic DNA oligonucleotide duplex. Due to its self-complementarity and because its structure is well established, the Dickerson-Drew dodecamer d(CGCGAATTCGCG) (DD12) was chosen [29]. It contains more G:C than A:T pairs, so according to the reports from the 1960s it should be affected by the presence of Ag^+^ ions. However, it is important to note that conclusions based on the investigation of polymeric DNA cannot necessarily be transferred to short oligonucleotides, as the latter have a larger share of terminal base pairs which tend to fray [30, 31], leading to different accessibility for metal ions. Nevertheless, as most current studies on the metal-binding properties of DNA make use of short oligonucleotides, their investigation in this respect is highly relevant.

## Materials and methods

### General

The Dickerson-Drew dodecamer DD12 [29] and the MOPS-d_15_ buffer were purchased from Eurogentec and EQ Laboratories GmbH, respectively, and used without any further purification.

### CD spectroscopy

CD spectra were recorded at a J-815 CD-spectrometer (Jasco) at 20 °C with a scan rate of 200 nm/min and a data interval of 0.1 nm with a five-fold accumulation. A baseline correction was applied by subtracting a blank measurement (buffer and NaClO_4_ solution only) from the measured values. During Ag^+^ titration, AgNO_3_ was added from an aqueous stock solution (1.25 mM) to the sample containing 5 mM MOPS (pH 6.8), 150 mM NaClO_4_, 2.5 µM dsDNA. CD spectra were analysed using OriginPro 2024 0.1.0.170 (OriginLab Corporation).

### NMR spectroscopy

NMR spectra were recorded on a Bruker AVANCE NEO 600 MHz spectrometer at 298.0 K, using a QCI cryo probe. Excitation sculpting for water suppression was used in 1D ^1^H and 2D-[^1^H,^1^H] NOESY NMR spectra (90% H_2_O/10 % D_2_O). 1D ^1^H NMR spectra were recorded with a relaxation delay of 2 s, 65536 points, and 128 scans using zgesgp or zg pulse programs. 1D ^31^P NMR spectra were recorded with composite pulse decoupling, using a 30° pulse, 2 s relaxation delay, 16384 points, and 1024 scans using the zgpg30 pulse program. 2D [^1^H,^1^H]-NOESY NMR spectra were recorded with a mixing time τ_m_ of 250 ms, 2 s relaxation delay and 4096×256 complex points.

[^1^H,^1^H]-TOCSY spectra necessary for the assignment of the resonance were recorded with a mixing time of 80 ms, 2 s relaxation delay, and 4096×256 complex points. [^1^H,^1^H]-DQF-COSY spectra necessary for the assignment of the resonance were recorded with a relaxation delay of 1.5 s, and 8192×256 complex points. ^1^H NMR chemical shifts were referenced externally to 0.5 mM 3-trimethylsilylpropane sulfonic acid (DSS) in water (δ_H_ = 0 ppm), and ^31^P NMR chemical shifts are reported relative to 85 % H_3_PO_4_ in water. NMR samples were prepared in H_2_O/D_2_O (10%) or D_2_O (100%) depending on the experiment with following concentrations: 0.5 mM dsDNA, 100 mM MOPS-d_15_ (pH 6.8), 150 mM NaClO_4_; 1.0 mM dsDNA, 100 mM MOPS-d_15_ (pH 6.8), 150 mM NaClO_4_; 2 mM dsDNA, 200 mM MOPS-d_15_ (pH 6.8), 150 mM NaClO_4_. Stock solutions of AgNO_3_ (50 mM) and NaCl (25 mM or 3.24 M) were prepared in D_2_O and added to the samples for titration. NMR spectra were processed and analysed using MestReNova 15.0.0 (Mestrelab Research) and Sparky (UCSF) software. The ^1^H NMR resonances of the DD12 duplex in the absence of Ag^+^ ions were assigned completely. The assignment is in agreement with that reported in the literature [32–34].

## Results and discussion

**Fig. 2:**
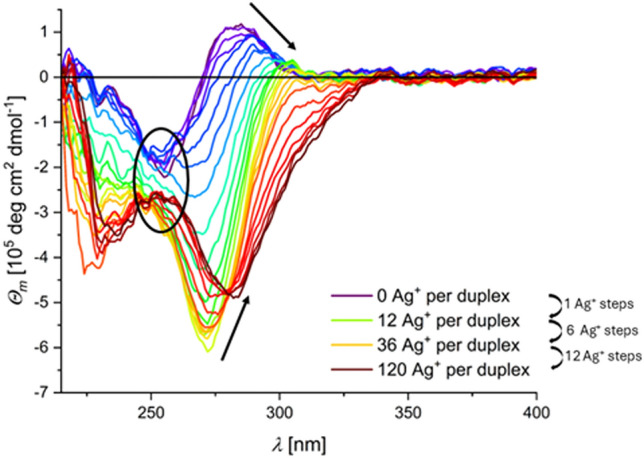
Set of CD spectra of the DD12 duplex with different amounts of Ag^+^ ions. The arrows point to the maxima and minima that change significantly with the addition of Ag^+^ ions and indicate the direction of the changes. The ellipse indicates the change from a local minimum to a local maximum. Experimental conditions: 5.0 µM dsDNA, 5 mM MOPS (pH 6.8), 150 mM NaClO_4_, AgNO_3_

A strong influence of excess Ag^+^ ions on the structure of DNA oligonucleotides was reported by several groups, irrespective of whether DNA duplexes with canonical base pairs [19], homo-base DNA oligonucleotides without the ability to form hydrogen-bond mediated base pairs [12, 24], DNA duplexes with a defined number of mismatches [26], or even DNA quadruplexes were investigated [35]. The binding of significant amounts of Ag^+^ ions to B-DNA results in the formation of a species with an increased melting temperature [36] and a characteristic CD-spectroscopic pattern (*vide infra*) [19, 25, 26]. This phenomenon is also observed for the DD12 examined here. With increasing equivalents of Ag^+^ ions, a drastic change is observed in the CD spectrum (Fig. 2). Such a change can only be due to a significant structural rearrangement of the DNA double helix. While the minimum at ca. 255 nm evolves into a maximum with increasing amounts of Ag^+^ (indicated by an ellipse in Fig. 2), the global minimum at ca. 270 nm and the global maximum at ca. 285 nm both shift to higher wavelengths (to ca. 280 nm and ca. 305 nm, respectively) and decrease in intensity (indicated by arrows). After the addition of 120 Ag^+^ ions per duplex, the CD spectrum shows only negative molar ellipticities above 220 nm, which is in good agreement with earlier studies (*vide supra*). This change in the oligonucleotide structure does not correlate linearly with the amount of Ag^+^ ions. Instead, only minor changes are observed in the CD spectrum up to the addition of 4 Ag^+^ ions per duplex. From 4 Ag^+^ ions per duplex onwards, the structural change per added Ag^+^ ion is more significant until approximately 12 Ag^+^ ions per duplex are added. This observation leads to the conclusion that a B-DNA-type duplex is still predominant for up to 4 Ag^+^ ions per duplex. Then, a significant structural change takes place upon the addition of further Ag^+^ ions until 12 Ag^+^ ions per duplex (i.e., one Ag^+^ per base pair) are present. Excess Ag^+^ only leads to gradual additional changes.

While the interaction of DNA oligonucleotides (with or without artificial chemically modified building blocks) with Ag^+^ ions has been widely studied [13, 19, 20], we still lack detailed knowledge of the initial binding sites of unmodified DNA duplexes towards Ag^+^ ions. Therefore, the question arises which building blocks of a DNA duplex are involved in the initial interaction between B-DNA and Ag^+^ ions or, to put it differently, which are the initial binding sites of a DNA duplex interacting with Ag^+^ ions. In addition to being relevant from a fundamental chemistry point of view, this knowledge is also important when it comes to the design of nucleic acids with Ag^+^-mediated base pairs involving artificial nucleobases [27], because once all pre-designed Ag^+^-binding sites are saturated, additional Ag^+^ will start interacting with the remaining parts of the DNA duplex. To answer this question, we chose NMR spectroscopy as a well-established powerful method to gain information on structures in solution, as widely applied including in bioinorganic contexts [37–39]. Hence, the interaction of the Dickerson-Drew dodecamer, which adopts a B-DNA duplex topology in solution [40, 41], with Ag^+^ ions was investigated by NMR spectroscopy. This work provides information about the interaction of Ag^+^ ions with nucleic acids and contributes to the general understanding of the interaction of nucleic acids with metal ions.

1D- and 2D-NMR experiments were performed at different ratios of Ag^+^:DNA. Most data were collected in the presence of three Ag^+^ ions per duplex because the CD spectra indicate very minor structural changes at this ratio (Fig. 2). The ^1^H NMR signals of the DD12 sequence in the absence of Ag^+^ ions were assigned completely and are in agreement with previous literature reports [32–34], and provide a fundament for assigning the changes upon the addition of Ag^+^ ions.

**Fig. 3:**
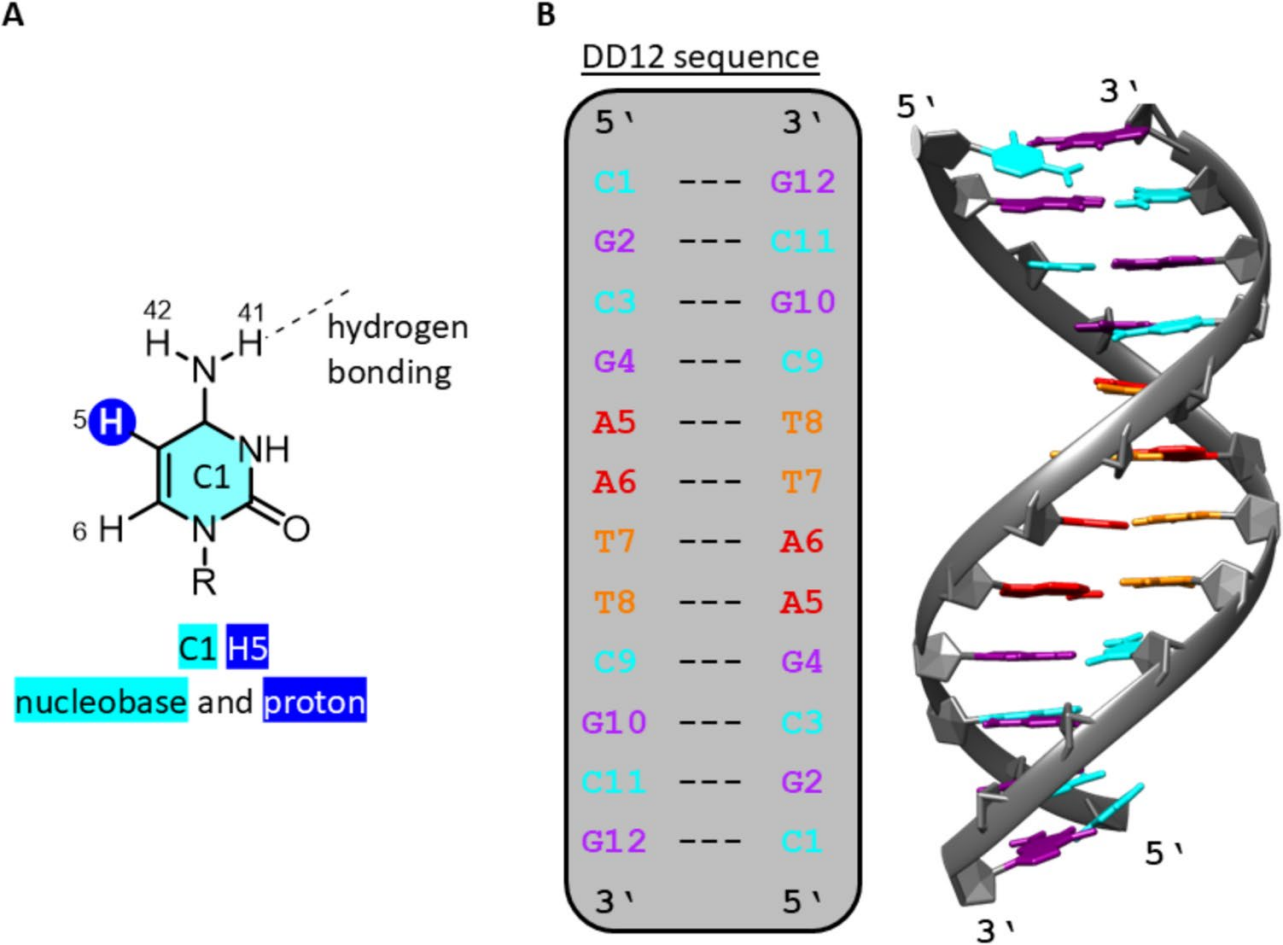
The numbering scheme is used for the nucleobase protons. **A** Proton 5 of cytosine 1 and **B** the Dickerson-Drew dodecamer sequence and its structure in solution. The figure was generated from PDB entry 2DAU [33] by using the program UCSF Chimera [42]

Due to the self-complementarity of the DD12 sequence, a symmetric structure is adopted and the number of NMR signals is reduced. The numbering scheme of the nucleobases (Fig. 3) used in this work includes the number of the respective nucleobase, indication of its location in the sequence, followed by the number of the proton based on the IUPAC numbering scheme for purine and pyrimidine bases [43].

### General coordination and release of Ag^+^ ions

**Fig. 4:**
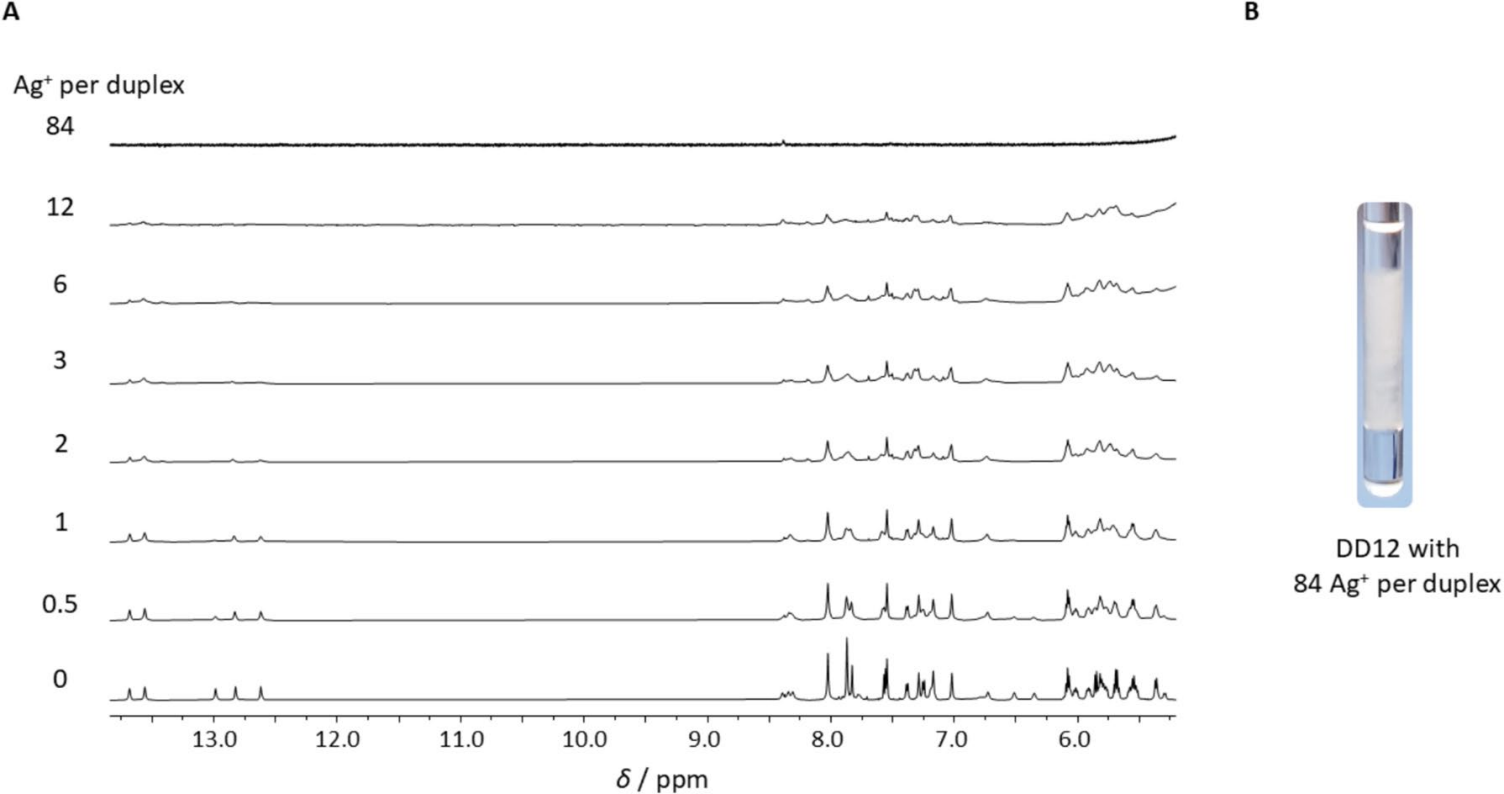
**A** ^1^H NMR spectra of DD12 in the presence of different amounts of Ag^+^ ions and **B** precipitation of DNA aggregates in the Shigemi NMR tube upon the addition of 84 Ag^+^ ions per duplex. Experimental conditions: H_2_O/10% D_2_O, 150 mM NaClO_4_, 0 – 12 Ag^+^ per duplex: 100 mM MOPS-d_15_ (pH 6.8), 0.5 mM dsDNA (**A**); 84 Ag^+^ per duplex: 200 mM MOPS-d_15_ (pH 6.8), 2 mM dsDNA (**A**, **B**)

As can be seen from the ^1^H NMR spectra of DD12, the signal intensity decreases drastically with increasing amounts of Ag^+^ ions (Fig. 4A). This results in an almost complete disappearance of ^1^H NMR signals for 12 Ag^+^ ions per duplex, i.e., one metal ion per base pair. Additionally, the signals are subject to line broadening which leads to a decrease in resolution and to a more difficult and less clear assignment. A qualitative comparison of the different spectra shows that three Ag^+^ ions per duplex represent the highest amount of Ag^+^ for which the NMR spectra still can be reasonably analysed. In the presence of more than 12 Ag^+^ ions per duplex, the oligonucleotides start to aggregate into larger structures, as can be deduced from the starting turbidity of the solution. In the presence of a larger excess of Ag^+^ ions, the larger oligonucleotide structures precipitate from the solution (Fig. 4B). Similar observations of precipitating DNA aggregates in the presence of Ag^+^ ions were published recently by Kondo *et al*. [19]. They identified the precipitate as Ag^+^-DNA rods interconnected *via* Ag^+^-mediated base pairs.

**Fig. 5:**
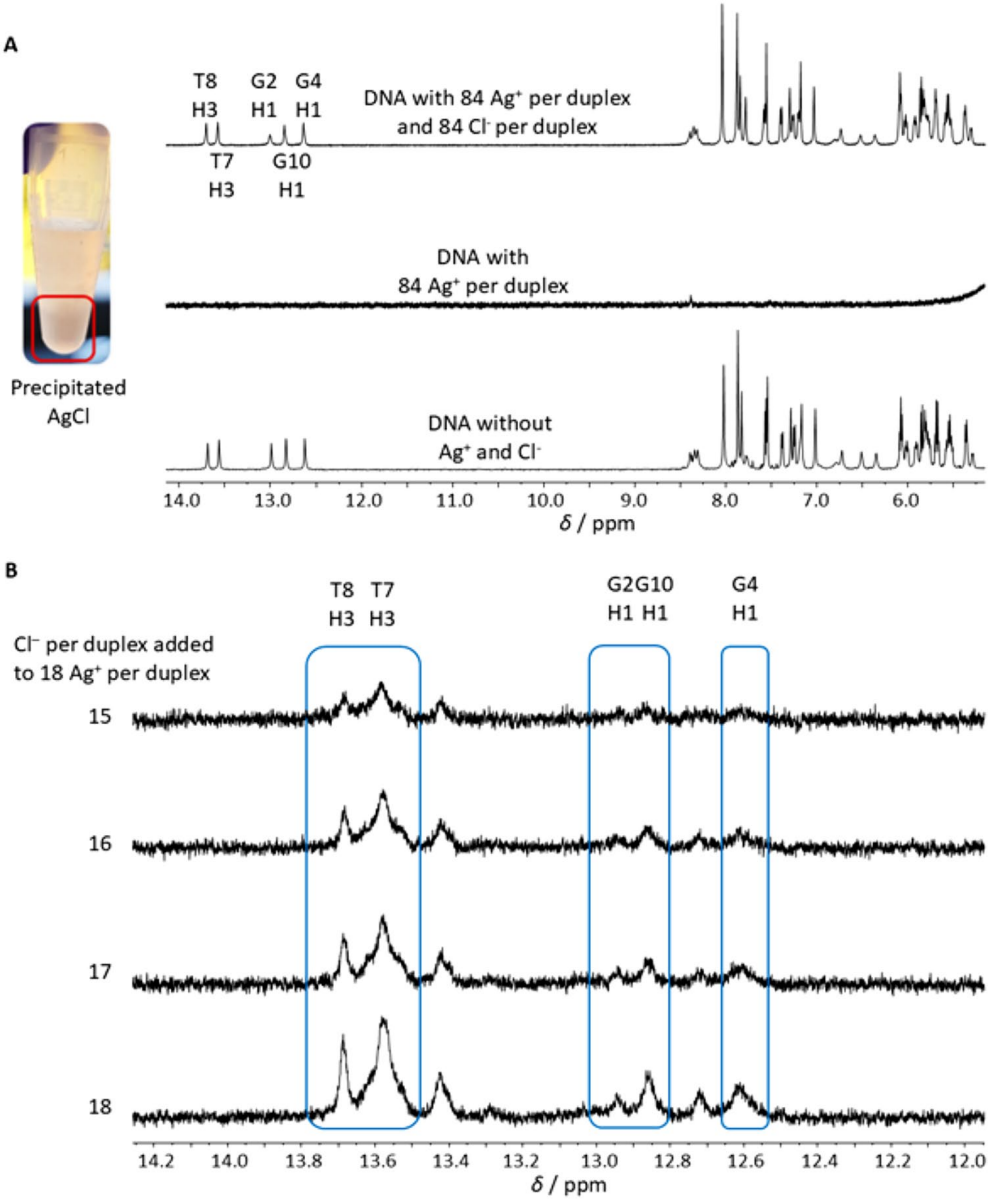
**A** Precipitated AgCl clearly visible by the naked eye and stacked ^1^H NMR spectra of DD12 before the addition of Ag^+^ ions, with 84 Ag^+^ ions per duplex, and the restored DD12 signals after the addition of Cl^–^. **B** Stepwise release of the Ag^+^ ions depending on the amount of added Cl^–^ and stepwise recovery of the original DD12 signals (marked by blue boxes). Only the chemical shift range of the imino protons is shown. Experimental conditions: H_2_O/10% D_2_O, 150 mM NaClO_4_, **A** 200 mM MOPS-d_15_ (pH 6.8), 2 mM dsDNA, **B** 100 mM MOPS-d_15_ (pH 6.8), 0.5 mM dsDNA

Due to the poor solubility of AgCl in water (solubility product of 1.8 · 10^–10^ mol^2^ L^–2^ [44]), the Ag^+^ ions bound to the DNA can be removed under the millimolar concentration conditions used in the NMR experiments *via* the addition of chloride ions, leading to an instantaneous precipitation of AgCl (Fig. 5). This indicates a rather loose binding of Ag^+^ ions to the DNA. In a similar way, chloride ions had been used to remove Ag^+^ ions from artificial imidazole–Ag^+^–imidazole base pairs within a DNA duplex [45]. The Ag^+^ ions can be removed stepwise, depending on the amount of chloride added to the solution. Even large amounts of Ag^+^ ions can be removed almost quantitatively from the DNA, and the DNA duplex signals in the ^1^H NMR spectrum are restored. This is not a general phenomenon: chloride ions are not necessarily able to remove Ag^+^ ions from silver-mediated base pairs. Seela *et al.* described for their investigated artificial metallo base pair (a homo base pair of the 8-aza-7-deazaadenine *N*^8^-2’-deoxyribonucleoside) that only iodide was able to remove the Ag^+^ ions completely. In contrast, chloride showed no effect on the Ag^+^-mediated base pair [36]. However, those experiments were performed at micromolar concentrations, so that precipitation was not expected based on the solubility product.

### Possible initial binding sites

In principle, nucleic acids provide various possibilities for an interaction with metal ions:

I. electrostatic interactions with the negatively charged sugar-phosphate backbone
II. coordination to the N7 position of purine bases
III. formation of metal-mediated base pairs *via* the Watson-Crick edge
IV. formation of metal-mediated base pairs *via* the Hoogsteen edge

Since the binding of Ag^+^ ions to B-DNA is based on several dynamic processes running in parallel, binding sites that are in principle known for Ag^+^ ions but occur less frequently and are therefore less relevant are not considered further. These include the coordination of Ag^+^ ions by keto groups or amino groups of cytosine and thymine, as described for a series of homo base pairs in the solid state [46]. In the following, we consider the four binding modes relevant to the solution as mentioned above and analyse the NMR spectroscopic data obtained from the titration of DD12 with Ag^+^ ions.

#### Electrostatic and non-specific binding to the phosphate groups of the nucleic acid backbone

Electrostatic binding of the Ag^+^ ions to the phosphate groups is expected to have a strong influence on the ^31^P NMR resonances (– 0.7 to – 1.9 ppm) and, to a lesser extent, on the ^1^H NMR resonances due to the deoxyribose hydrogen atoms. The binding of metal ions should withdraw electron density and thus cause a downfield shift of the resonances. Since the phosphate backbone extends over the entire strand and all phosphate residues should be equally accessible, non-specific binding of the Ag^+^ ions would be expected. Given the presence of only three Ag^+^ ions per DD12 duplex, this translates into a statistical distribution over the backbone and therefore all affected signals should be shifted to a similar extent. The ^1^H NMR resonances associated with the nucleobases should only be marginally influenced.

**Fig. 6:**
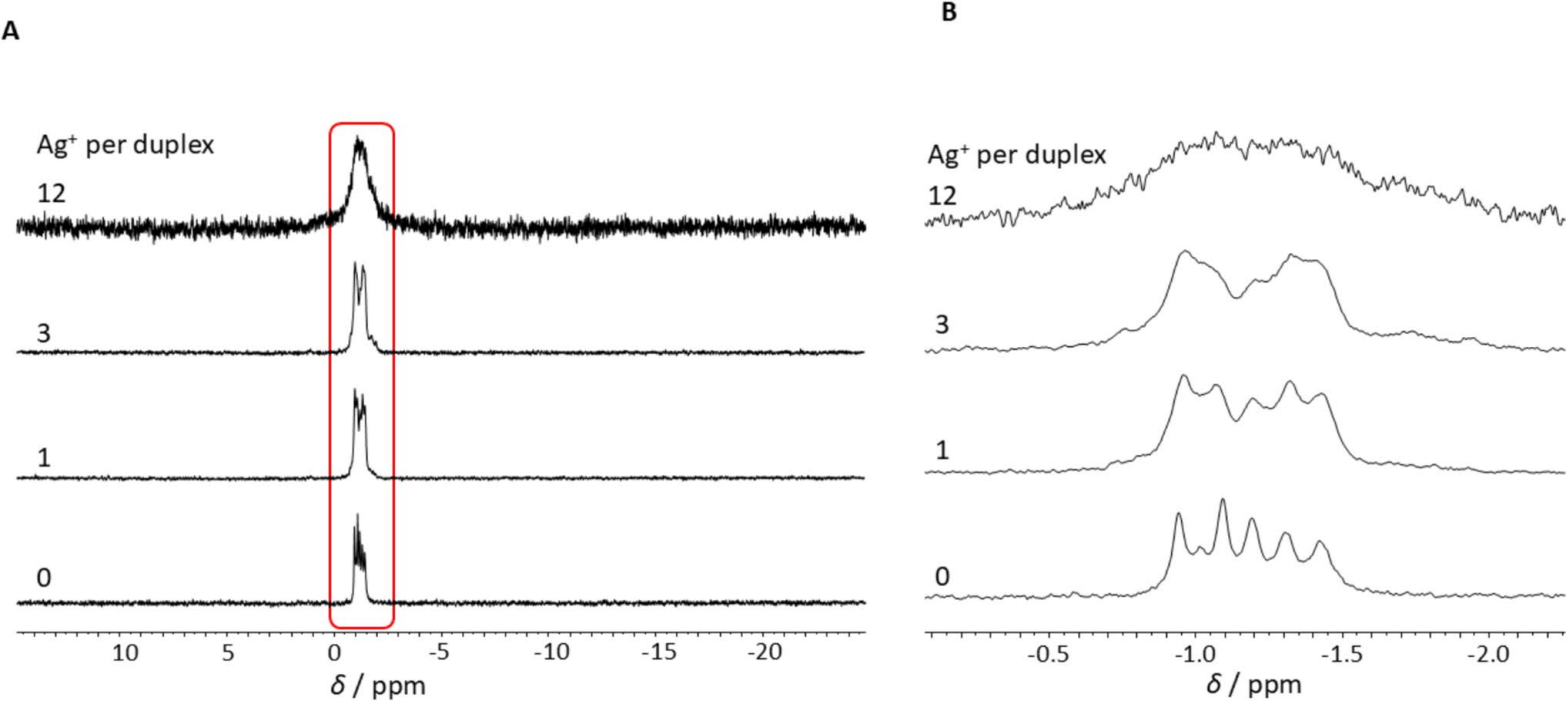
**A** Stacked ^31^P NMR spectra of DD12 in the presence of increasing amounts of Ag^+^ ions and **B** detail of a selected region (red box) of the ^31^P NMR spectra. Experimental conditions: H_2_O/10% D_2_O for 1 – 12 Ag^+^ per duplex; D_2_O for 0 Ag^+^ per duplex, 150 mM NaClO_4_, 100 mM MOPS-d_15_ (pH 6.8), dsDNA: 1 mM for 0 – 3 Ag^+^ per duplex; 0.5 mM for 12 Ag^+^ per duplex

As can be seen from Fig. 6, the ^31^P NMR resonances of the DNA are not influenced significantly by the addition of Ag^+^ ions. When compared to the spectrum in the absence of Ag^+^ ions, no significant change in the chemical shifts or dominant appearance of a second set of signals at different chemical shift is observed. This is not surprising, as the binding of soft metal cations such as Ag^+^ by soft donor atoms, such as nitrogen, is favoured [13]. However, a decreasing signal intensity and line broadening should be noted. As a result, only a single broad signal is detected upon the addition of 12 Ag^+^ ions per duplex. This observation is consistent with the general trend of line broadening and loss of signal intensity in the presence of Ag^+^ ions. Nevertheless, a low and broad signal set centring around –1.8 ppm appears upon the addition of Ag^+^ ions with very low but increasing intensity with increasing amounts of Ag^+^ ions. As will be shown in the following, the binding of Ag^+^ ions to DD12 is subject to several subprocesses running in parallel and contributes to the overall binding process to varying degrees. Due to the high complexity, this work will focus on major contributions only.

**Fig. 7:**
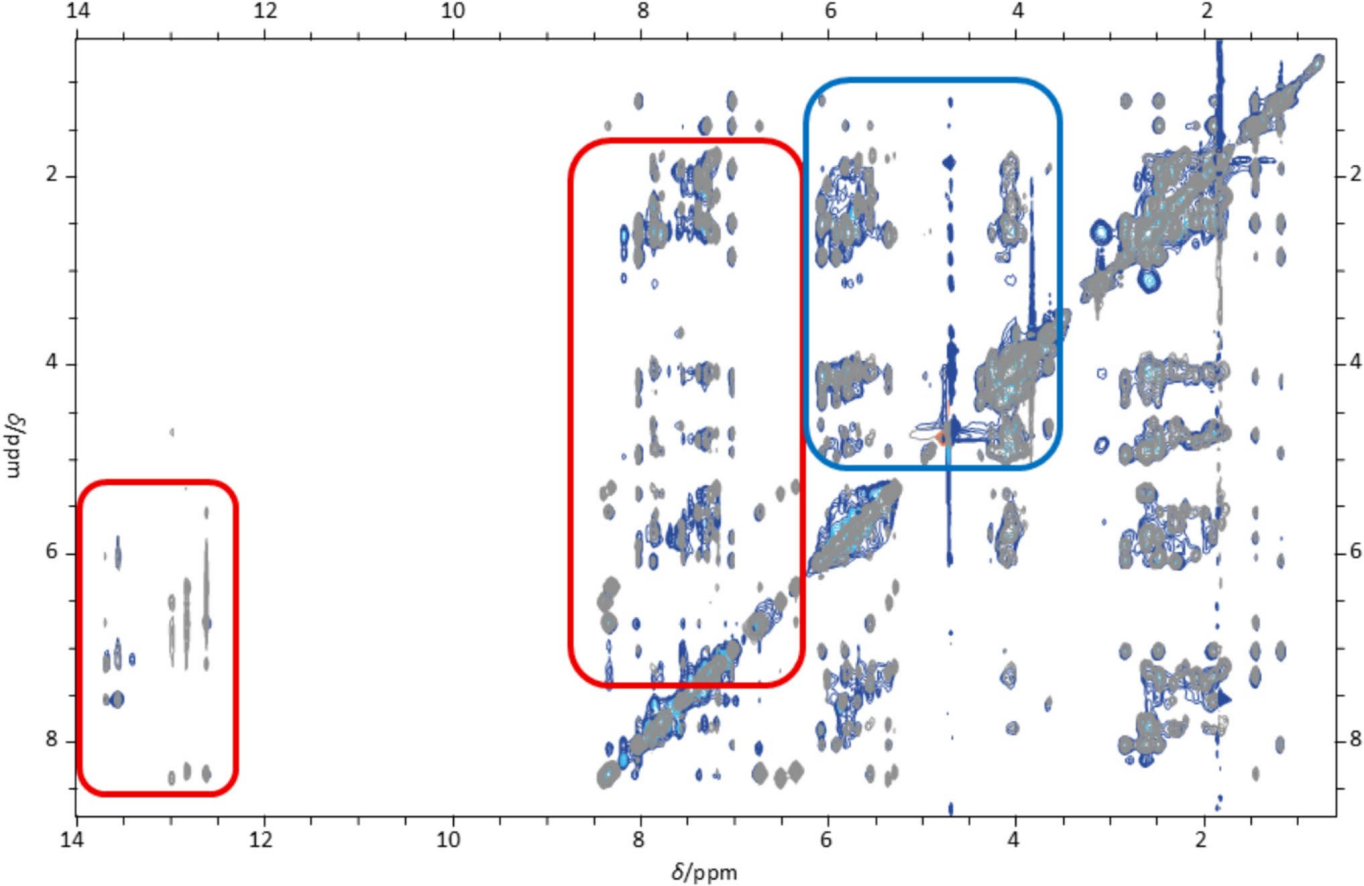
Overlay of [^1^H, ^1^H] NOESY NMR spectra of 0 (grey) and 3 Ag^+^ ions per duplex (blue gradient). More significant changes are observed in the regions of cross-peaks involving nucleobase protons (red box) than in the regions of cross-peaks involving sugar protons only (blue box). Experimental conditions: H_2_O/10% D_2_O, 100 mM MOPS-d_15_ (pH 6.8), 150 mM NaClO_4_, 1 mM dsDNA

Further evidence against the hypothesis of electrostatic binding of Ag^+^ ions to the phosphate backbone is provided by the significant changes observed for the ^1^H NMR signals of the nucleobases upon the addition of Ag^+^. These changes include disappearance of signals, the shift of signals, and the appearance of a second set of signals. Figure 7 compares the [^1^H,^1^H] NOESY NMR spectra of DD12 in the absence (grey) and the presence of three Ag^+^ ions per duplex (blue gradient). It visualises that the nucleobase proton resonances undergo significant changes in chemical shift, while those of the sugar protons are less affected. The most intense effect of the addition of Ag^+^ on the resonances is observed for the sugar protons H2’ and H2’’, some of them show the appearance of additional cross peaks with the aromatic protons of the nucleobases in the presence of three Ag^+^ ions per duplex, which unfortunately cannot be assigned unequivocally to particular nucleobases due to broad and overlapping signals. Marginal changes in the sugar pucker, which might be induced by the larger steric bulk of an Ag^+^ ion compared to a proton, might be responsible for these new signals. Hence, the appearance of additional signals for a few nucleobases does not necessarily indicate the direct coordination of Ag^+^ ions by the sugar-phosphate backbone but might result from an altered sugar pucker due to the coordination of Ag^+^ ions to nearby binding sites (*vide infra*).

Taking into consideration the HSAB concept, it is quite unlikely that soft cations such as Ag^+^ ions are electrostatically bound by the negatively charged phosphate backbone as the endocyclic heteroatoms of the nucleobases represent softer and readily available coordination sites. According to literature reports, Ag^+^ ions do not interact with either the phosphate backbone or the sugar residues. Coordination occurs exclusively through the nucleobases [47].

#### Non-specific binding to sterically accessible nucleobase donor atoms, typically purine N7 in the major groove

**Fig. 8:**
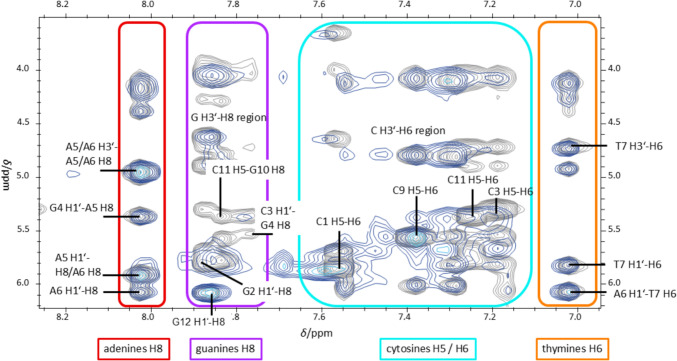
Overlay of [^1^H, ^1^H] NOESY NMR spectra of 0 (grey) and 3 Ag^+^ ions per duplex (blue gradient). H8 protons of adenine (red box) and guanine (purple box) residues do not show significant changes. In contrast, cytosine residues H5 and H6 (cyan-coloured box) with significant changes and thymine residues H6 (orange box) with preserved original signals. Experimental conditions: H_2_O/10% D_2_O, 100 mM MOPS-d_15_ (pH 6.8), 150 mM NaClO_4_ and 1 mM dsDNA

Other possible initial binding sites are the sterically accessible donor atoms of the nucleobases. Coordination of Ag^+^ ions by the N7 nitrogen atom of purine bases has been reported in the literature for both artificial and natural nucleobases in model complexes [47]. A coordination by the N7 atom, typically accessible *via* the major groove, is expected to result in a statistical contribution over all purines of the duplex and hence should lead to similar chemical shift changes for the H8 protons of all purine residues (per single strand are 4 × guanine and 2 × adenine present). As Ag^+^ ions coordination is labile and hence is a dynamic process, the binding of Ag^+^ ions to the N7 donor atoms should result in an overall slight change of chemical shift of the ^1^H NMR resonances. Interestingly, a comparison of the [^1^H,^1^H] NOESY NMR spectra at 0 and 3 Ag^+^ ions per duplex shows that the H8 protons of guanine and adenine residues are hardly affected by the addition of Ag^+^ ions (Fig. 8, red and purple box). However, the signals assigned to the protons H5 and H6 of cytosine moieties are broadened and slightly shifted downfield, meaning that these protons other than purine H8 are clearly influenced (Fig. 8, cyan blue box). The fact that the chemical shifts of the H1’ and H3’ protons do not change significantly indicates that the Ag^+^ ions are located closer to the nucleobases than to the deoxyribose moieties (Fig. 8).

From a geometric perspective, Ag^+^ ions prefer the coordination by two ligands in a linear fashion. Taking into consideration that coordination of Ag^+^ ions by nitrogen donor atoms is expected instead of by oxygen atoms of the keto groups, a linear coordination environment is not supported in the major groove without significant conformational changes of the helical duplex structure. Such a change would become manifest in the CD spectrum, which is not the case upon the addition of three Ag^+^ ions per duplex (Fig. 2). Hence, while a binding of the Ag^+^ ions to the nucleobases from the major groove cannot be excluded entirely, it is unlikely to be the relevant binding mode.

#### Formation of Ag^+^-modified base pairs by replacing a proton within a Watson-Crick pair

Nucleobases are able to form so-called metal-modified base pairs by formally substituting a hydrogen bond in a Watson-Crick base pair by coordinate bonds to a metal ion [7]. These are known for various combinations of nucleobases and metal cations, including a Ag^+^-modified guanine cytosine base pair (G–Ag^+^–C) [19].

At the onset of the titration of DD12 with Ag^+^ ions, only minor changes are observed in the CD spectrum. The changes become significant in the presence of more than three Ag^+^ ions per duplex. In the case of the formation of metal-modified base pairs upon coordination of Ag^+^ ions, large changes in the CD spectrum are not expected as the structure of the double helix should essentially be preserved [47]. The absence of large changes in the CD spectrum is not an unequivocal indication for the formation of metal-modified base pairs, as this phenomenon is also expected for non-specifically bound Ag^+^ ions, potentially interacting with bound Ag^+^ ions, either by phosphate groups or donor atoms exposed in the major group. However, these two binding modes were deemed to be insignificant based on the NMR data to be the dominating processes (*vide supra*). In the following, we will take a closer look at the resonances of various protons of the nucleobases and their changes upon the addition of Ag^+^ ions.

**Fig. 9:**
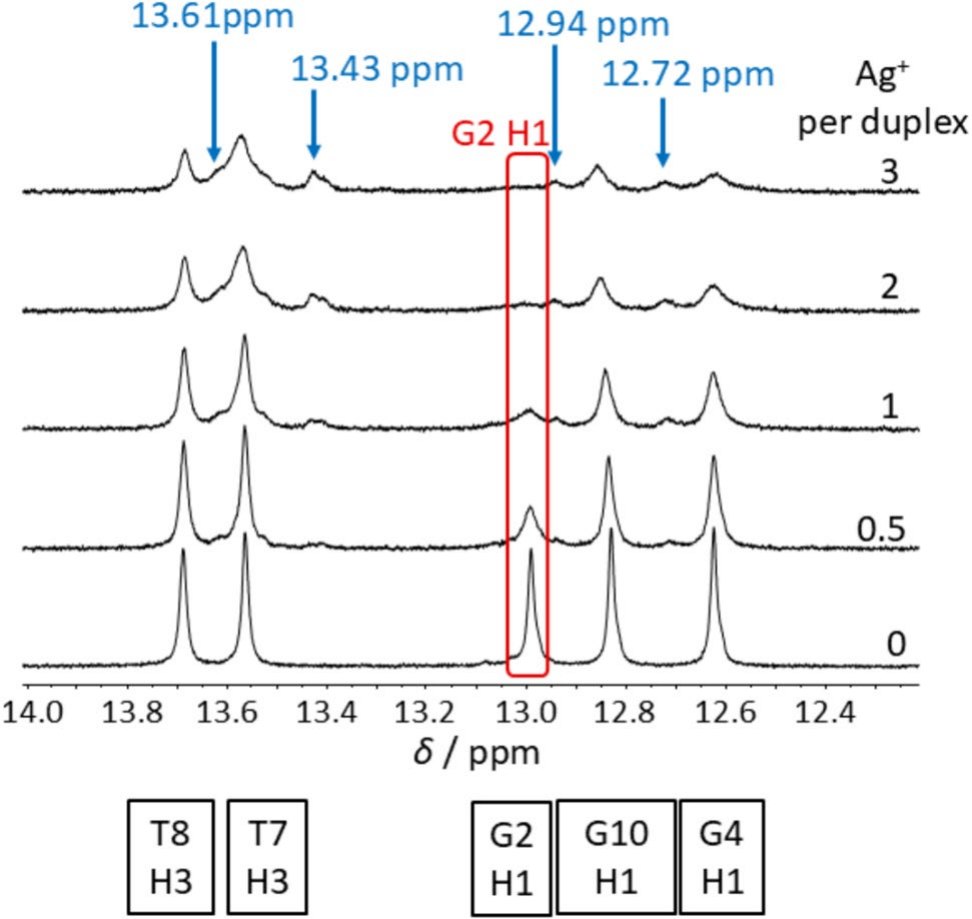
Imino proton region of ^1^H NMR spectrum of DD12 in the presence of different amounts of Ag^+^ ions. The signal of G2 H1 disappears (red box) with increasing amounts of Ag^+^ ions while new signals appear (blue arrows). Experimental conditions: H_2_O/10% D_2_O, 100 mM MOPS-d_15_ (pH 6.8), 150 mM NaClO_4_ and 0.5 mM dsDNA

The H1 imino proton of the terminal guanine residue G12 and the H41 amino proton of its complementary cytosine nucleobase C1, both of which are involved in the formation of one hydrogen bond each, are not observed by NMR spectroscopy due to the fraying of the double helix and concomitant breaking of the hydrogen bonds, resulting in a rapid exchange with the solvent protons. Hence, only three of the four G:C base pairs can be observed by ^1^H NMR spectroscopy, namely, G2:C11, C3:G10 and G4:C9. A more detailed examination of the imino proton region of the ^1^H NMR spectra (14.0 – 12.0 ppm) reveals that the signals of the nucleobases thymine and guanine are already affected by the presence of 0.5 Ag^+^ ions per duplex (Fig. 9). Nevertheless, the signals change to a different extent, e.g., G2 H1 (12.99 ppm, red box in Fig. 8) exhibits the most sensitive behaviour towards silver ion addition.

As the formation of a G–Ag^+^–C metal-modified base pair (Fig. 1C) involves the replacement of the H1 proton by an Ag^+^ ion, the disappearance of its ^1^H NMR resonance could serve as an indicator for the formation. This is observed for G2 H1. In the presence of three Ag^+^ ions per duplex, its signal at 12.99 ppm disappears. Likewise, the guanine bases G10 and G4 are strongly affected by one Ag^+^ ion per duplex and beyond, as reflected in a decrease in the H1 signal intensity as well as a slight shift of the G10 H1 resonance from 12.83 ppm to 12.86 ppm and the appearance of a new signal for the G4 H1 proton at 12.72 ppm. A further new signal at 12.94 ppm (only visible in the 1D ^1^H NMR spectra, possibly due to sufficient intensity only in the 1D NMR spectra), cannot be assigned.

In contrast, the resonances of the imino protons of the thymine bases T7 and T8 undergo a comparatively minor change (Fig. 9). It should be emphasized that here two new broad signals emerge at 13.61 and 13.43 ppm. In contrast to the signal at 13.61 ppm, for which no cross-peaks can be identified in the [^1^H,^1^H] NOESY spectrum, the more pronounced signal at 13.43 ppm shows cross peaks to the original signals of T7 H3 and T8 H3, indicating an equilibrium between two structures. Given the high sensitivity of exchangeable protons to structural modifications and variations in solution conditions, it is not surprising that new signals corresponding to thymine imino protons are observed, even though their non-exchangeable proton signals remain largely unaltered. As a result of Ag^+^ ions coordination to the terminal base pairs, a change in the solvent accessibility of inner base pairs is anticipated, even if the overall conformation at these internal positions remains unchanged.

**Fig. 10:**
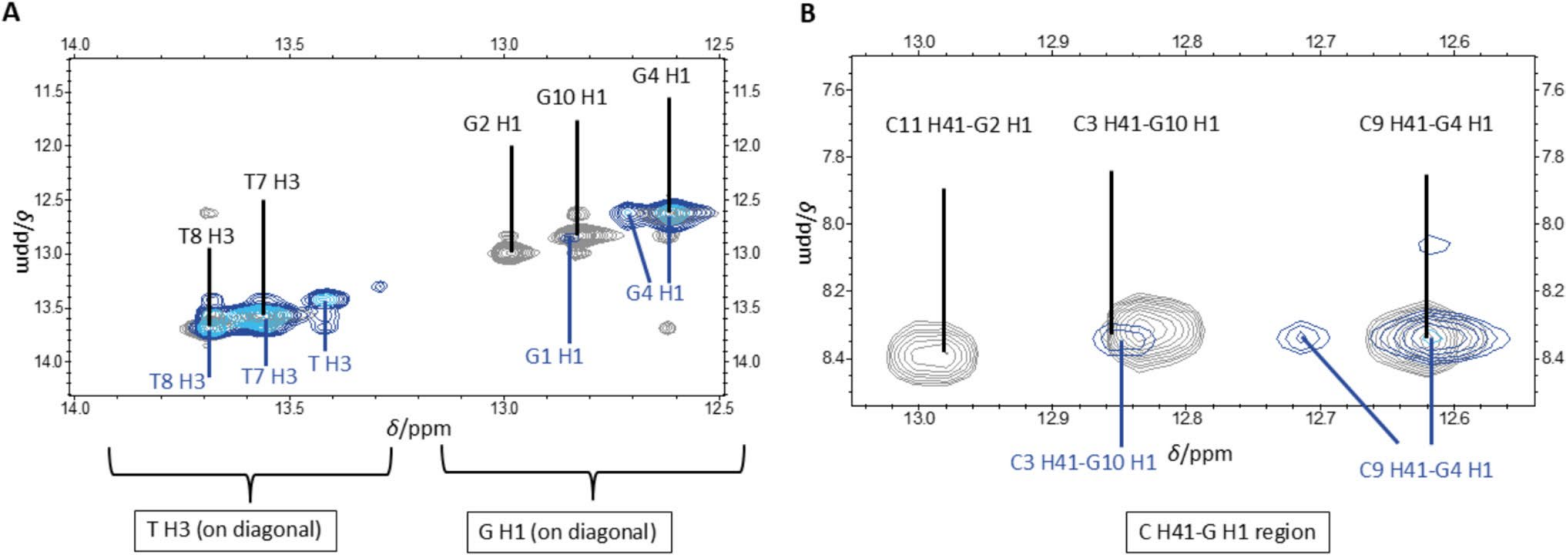
Overlay of [^1^H,^1^H] NOESY NMR spectra of 0 (grey) and 3 Ag^+^ ions per duplex (blue gradient). **A** Diagonal of imino protons T H3 and G H1 and **B** cross peaks of imino protons G H1 with amino protons C H41 involved in hydrogen bonding. Experimental conditions: H_2_O/10% D_2_O, 100 mM MOPS-d_15_ (pH 6.8), 150 mM NaClO_4_, 1 mM dsDNA

By breaking the hydrogen bonds of a G:C base pair, the cytosine amino protons H41 and H42 are no longer chemically distinguishable and consequently lead to a single signal in the NMR spectrum, which should have a chemical shift similar to that of the original proton H42 (around 6.8 – 6.3 ppm) not involved in the hydrogen bond. For the G2-complementary nucleobase C11, the G2 H1/C11 H41 and C11 H41/C11 H42 cross peaks disappear in the presence of three Ag^+^ ions per duplex (Fig. 10), which indicates the breaking of the hydrogen bonding.

**Fig. 11:**
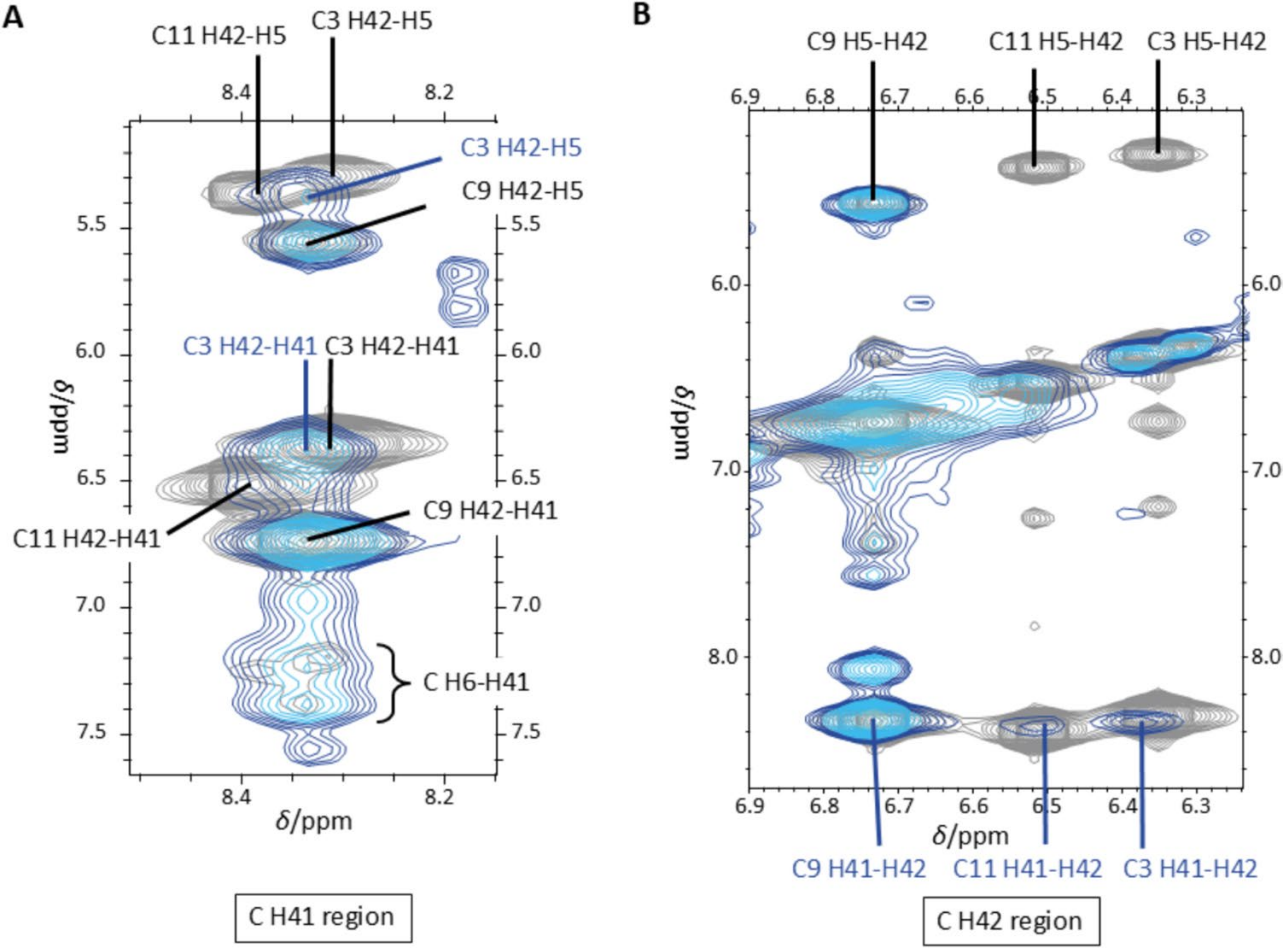
Overlay of [^1^H,^1^H] NOESY NMR spectra of 0 (grey) and 3 Ag^+^ ions per duplex (blue gradient) showing the cytosine amino protons **A** H41 involved in a hydrogen bond and **B** H42 not involved in a hydrogen bond. Experimental conditions: H_2_O/10% D_2_O, 100 mM MOPS-d_15_ (pH 6.8), 150 mM NaClO_4_, 1 mM dsDNA

The cytosine amino protons C3 H41 (8.31 ppm shifting to 8.34 ppm) and C3 H42 (6.35 ppm shifting to 6.39 ppm) show fewer cross peaks in the presence of three Ag^+^ ions per duplex (Fig. 11). These cross peaks of C3 amino protons are slightly shifted to a lower field and are less intense than the original signals, e.g., C3 H42-C3 H41. In contrast to the other cytosine residues, the amino protons C9 H41 and C9 H42 remain intense signals at their respective original chemical shifts also in the presence of three Ag^+^ ions per duplex. Here, only a broadening of the cross peaks is observed. This shows that C3, which is closer to the strand’s end, is significantly more influenced by the added Ag^+^ ions than C9 which is consistent with the observations on the imino proton NMR signals of the guanines described above.

The aforementioned changes of the signals of the guanine imino protons and the cytosine amino protons indicate that G2 H1 was substituted and that the hydrogen bonds of the G2:C11 pair were broken, suggesting a possible formation of an Ag^+^-modified base pair *via* the Watson-Crick edge at this position upon the addition of three Ag^+^ ions per duplex.

#### Ag^+^-mediated base pair formation via the Hoogsteen edge

**Fig. 12:**
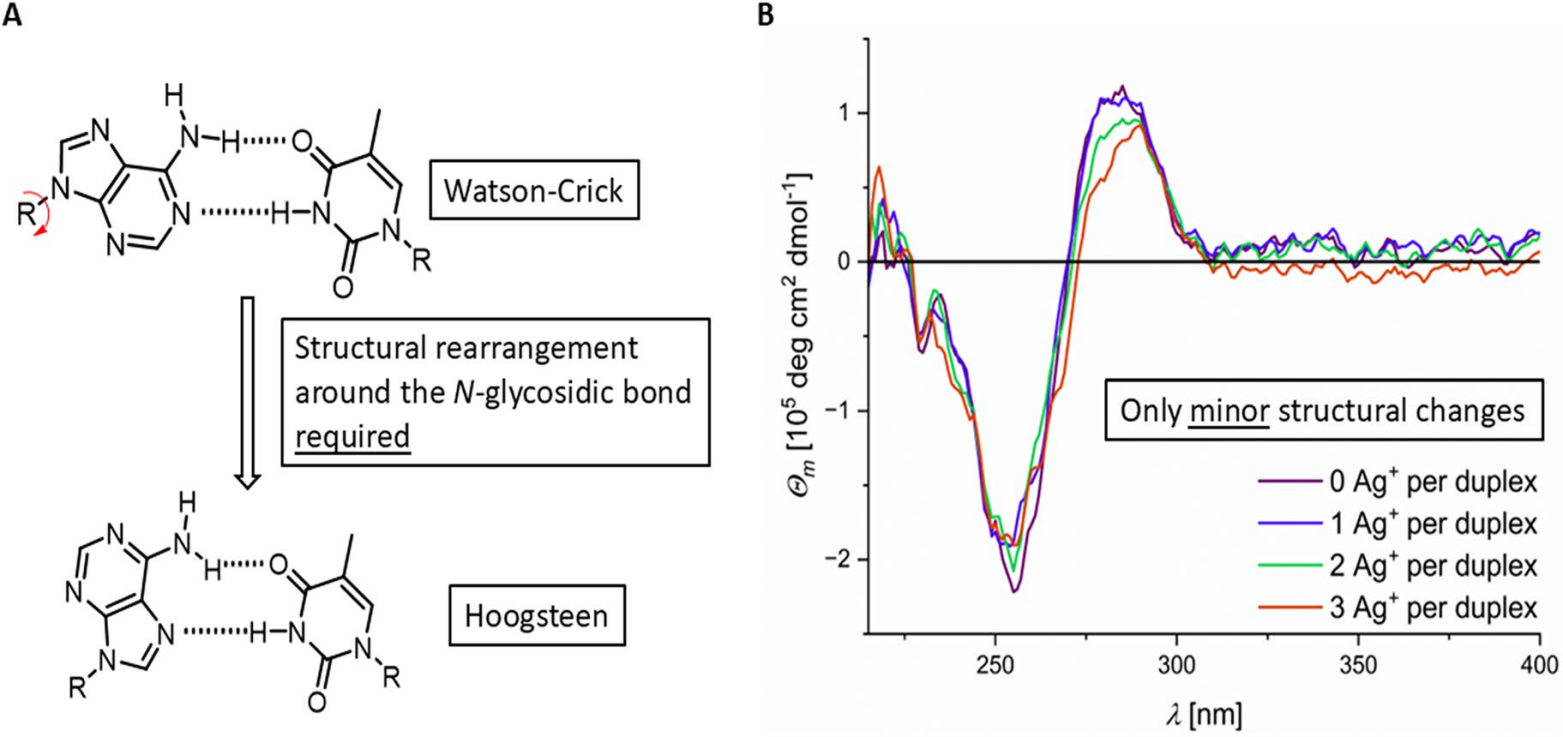
**A** Structural rearrangement around the *N*-glycosidic bond to allow Hoogsteen-type base pairing of guanine and cytosine and **B** only minor structural changes in the CD spectrum of DD12 for 0 – 3 Ag^+^ ions per duplex. Experimental conditions: 5 mM MOPS (pH 6.8), 150 mM NaClO_4_, 5.0 µM dsDNA

Assuming that Ag^+^-mediated base pairs may also be formed *via* the Hoogsteen edge, structural changes of the double helix would be expected as reorientation of the purine bases around the *N*-glycosidic bond would be required (Fig. 12A). Such changes should lead to changes in the CD spectrum compared to canonical B-DNA. With regard to the identification of the initial binding sites, at small amounts of Ag^+^ ions (three Ag^+^ ions per duplex) the CD spectrum shows only minor changes (Fig. 12B), allowing us to conclude that a coordination *via* the Hoogsteen edge would contribute only marginally to the overall binding of Ag^+^ ions. For more than three Ag^+^ ions per duplex, significant structural changes are confirmed CD spectroscopy, indeed suggesting that a structural reorientation becomes a relevant process in the presence of larger amounts of Ag^+^ ions (Fig. 2).

Moreover, the formation of one (or a few) Ag^+^-mediated base pair(s) without any site-specificity would result in the generation of a range of different DNA species, which would in turn yield a complex NMR spectrum with low-intensity signals. Accordingly, the addition of more than one Ag^+^ ion per base pair leads to the formation of various different species, as confirmed by the complex NMR spectra which cannot be clearly assigned. The formation of larger DNA aggregates is evident from precipitation starting in the presence of 12 Ag^+^ ions per duplex.

From this, we can conclude that the formation of non-Watson-Crick-type metal-mediated base becomes more relevant at larger amounts of Ag^+^ ions, i.e., for more than three Ag^+^ ions per duplex. This is in good agreement with the formation of an Ag^+^-DNA rod of a very similar DNA sequence comprising non-canonical Ag^+^-mediated base pairs, as observed in the solid state and in solution [19]. These Ag^+^-DNA rods show a CD spectrum very similar to the one observed for DD12 in the presence of excess Ag^+^ ions. Additionally, their NMR signals become broad and of low intensity with increasing amounts of Ag^+^ ions [19].

### Localisation of Ag^+^ ions along the double-helical B-DNA

After concluding that the formation of Ag^+^-modified G–Ag^+^–C base pairs represent the most likely initial mode of Ag^+^ binding, the localisation of the Ag^+^ ions along the B-DNA duplex is of interest as well. In principle, the Ag^+^ ions could use different pathways to enter the duplex, namely from the termini, from the major groove, or a combination of both. For the latter two options, a slight change of several resonances would be expected.

Upon inspection of the [^1^H, ^1^H] NOESY NMR spectra of DD12 in the presence of 0 and 3 Ag^+^ ions per duplex, respectively, significant changes in the NMR signals of guanine proton H8 and cytosine protons H5 and H6, including line broadening and shifted signals, lead to the assumption that guanine and cytosine residues are more affected by the Ag^+^ binding than thymine and adenine residues (Fig. 8). The latter show significantly less chemical shift perturbation. It is, therefore, anticipated that coordinated Ag^+^ ions will be predominantly located in the vicinity of the G:C base pairs. When comparing the ^1^H NMR spectra for different ratios of DD12:Ag^+^, the signals of the guanine imino protons are affected to a varying degree (Fig. 9). Already upon addition of less than three Ag^+^ ions per duplex, the resonance of the G2 H1 imino proton at 12.99 ppm is affected significantly, resulting in a decreasing signal intensity upon Ag^+^ ions addition and a complete disappearance of the signal at three Ag^+^ ions per duplex. Furthermore, the resonances of the cytosine protons H41 and H42 amino protons are influenced to a varying degree (Fig. 11), with the change decreasing from C11 *via* C3 to C9, i.e., from the outer to the inner residues. While the cross peaks involving C11 disappear almost completely, the original signals are still visible with high intensity for C9. This observation supports the hypothesis that Ag^+^ ions initially enter from the termini into the interior of the double helix, where they become coordinated within the helix. The fact that the resonances are influenced to a different extent eliminates the possibility of Ag^+^ ions preferentially localizing in the major groove or entering the duplex *via* the major groove. The entry from the termini into the duplex is feasible, given that the outermost base pairs are the more accessible and flexible regions of the helix, less constrained by the rigidity of the duplex and thus providing sufficient space to accommodate slight distortions required for metal ion coordination. At higher concentrations of Ag^+^ ions, when binding within the internal nucleobases of the DNA strand becomes necessary, the structure is likely to undergo refolding, resulting in the altered patterns observed in CD spectra.

**Fig. 13:**
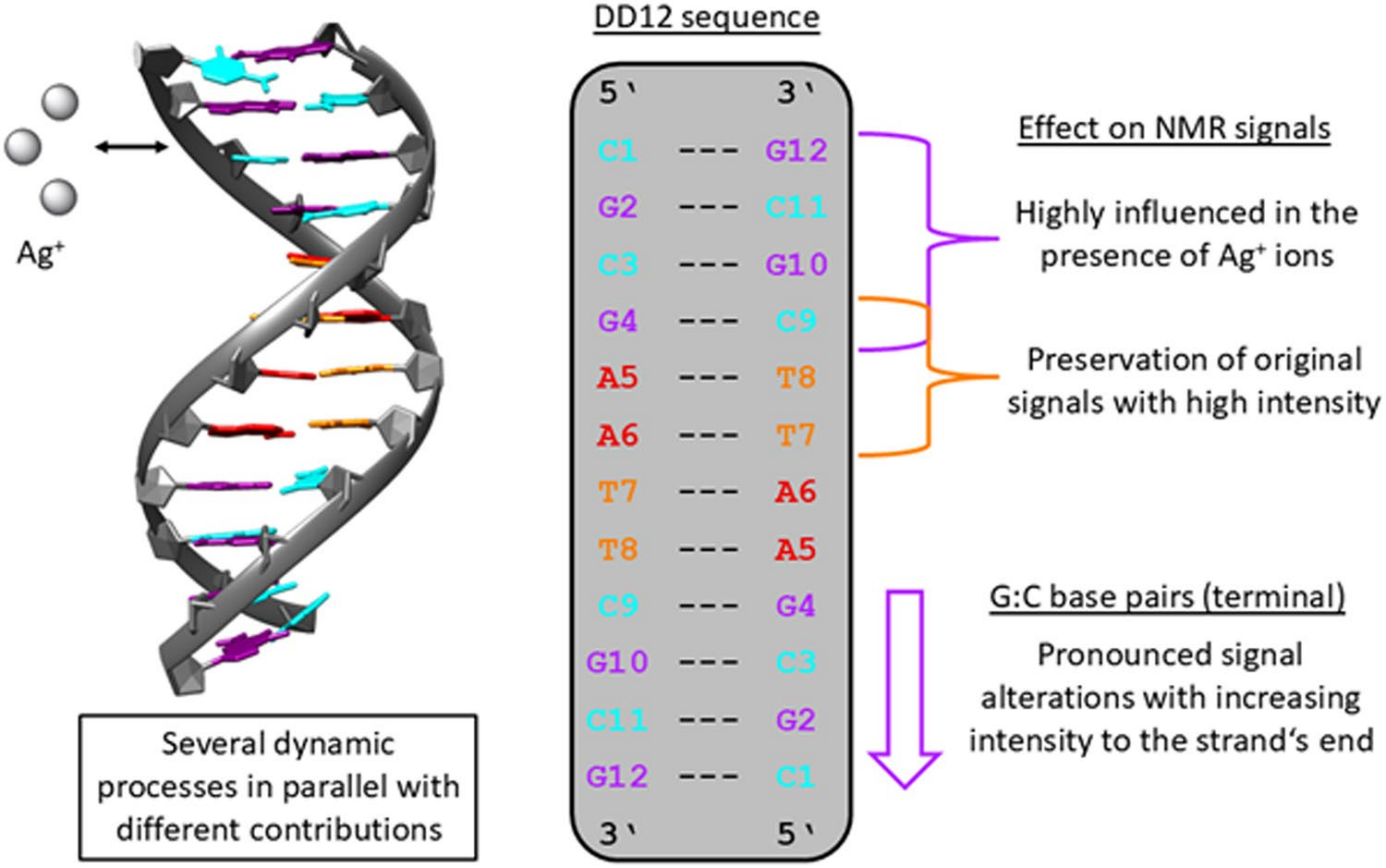
Coordination of Ag^+^ ions by B-DNA includes several dynamic processes in parallel with different contributions to the overall phenomenon. The NMR resonances of the outer G:C base pairs are significantly influenced in the presence of Ag^+^ ions while the original signals of the A:T base pairs in the interior of the helix are preserved. The outer G:C base pairs are influenced to a larger extent than the inner ones. The figure was generated from PDB entry 2DAU [33] using the program UCSF Chimera [42]

Taking into consideration the more significant changes for for outer G:C base pairs, it can be concluded that the Ag^+^ ions are initially preferentially bound by the outer nucleobases, guanine and cytosine, within the helix (Fig. 13). However, it needs to be kept in mind that different coordination processes may take place in parallel with different contributions to the overall binding.

The determination of a specific binding site, in the sense of an explicit donor atom, of the Ag^+^ ions would be an overinterpretation of the present NMR data. It would require further experiments, such as using isotopically labelled nucleobases, chemically modified deazapurine variants or variations in the sequence. Nevertheless, it needs to be emphasized that the NMR and CD data indicate the specific coordination by the nucleobases between the two single strands as the dominating initial binding process, with the Ag^+^ ions entering from the termini into the duplex. Hence, the formation of Ag^+^-modified G–Ag^+^–C base pairs *via* the Watson-Crick edge is the most likely process.

## Conclusions

Based on a series of NMR spectra of the DD12 duplex in the presence of different amounts of Ag^+^ ions, four conclusions can be drawn with respect to the objective to determine the initial Ag^+^ ion binding sites of unmodified B-DNA oligonucleotide duplexes. First, the NMR data indicate that Ag^+^ ions are bound by the nucleobases. Several processes appear to occur in parallel. According to only minor changes in the CD spectrum and based on the loss (or shift) of signals of the guanine G2 and G10 imino protons, the formation of Ag^+^-modified G–Ag^+^–C Watson-Crick base pairs should be the dominating process at sub stochiometric amounts of three Ag^+^ ions per duplex. Second, coordination takes place within the duplex *via* the terminal nucleobases, as concluded from the more significant changes of the NMR signals for the outer G:C base pairs compared to the inner ones. Third, the bound Ag^+^ ions can be released easily by the addition of chloride anions, demonstrating the loose coordination of Ag^+^ ions by an unmodified DNA duplex. Fourth, the addition of almost stoichiometric amounts of Ag^+^ ions results in the formation of larger DNA structures which start to precipitate from the solution and show significant changes in CD spectra.

These conclusions in part deviate from those drawn in the 1960s based on spectrophotometric and potentiometric studies but do not necessarily contradict them, as different experimental conditions and, more importantly, different DNA sequences were used. Importantly, the formation of metal-modified base pairs *via* the formal substitution of a proton within a hydrogen bond by an Ag^+^ ion was proposed in the 1960s [14] and is reinforced with the present study.

To elucidate the specific donor atoms involved in the coordination and the binding processes in more detail, it will be essential to label individual nucleobases with NMR-active isotopes such as ^15^N or apply deazapurine derivatives. Another aspect to be investigated next are the sequence dependence and the interaction with other (diamagnetic) metal cations, such as Cu^+^ and Hg^2+^, which are able to form metal-mediated base pairs as well [48, 49].

In summary, we could show that initially Ag^+^ ions prefer the bind inside the B-DNA duplex, entering the helix from the fraying ends and potentially forming G–Ag^+^–C base pairs. Moreover, we could exclude other possible binding sites, such as the phosphate backbone [11], contributing to the general understanding of the interaction of nucleic acids with metal ions. Once a certain amount of Ag^+^ ions is present, entirely new structures are adopted, likely to involve Ag^+^-mediated base pairs also *via* the purine N7 positions [19].

## Data Availability

Data is provided within the manuscript.
